# Determinants of the postprandial triglyceride response to a high-fat meal in healthy overweight and obese adults

**DOI:** 10.1186/s12944-021-01543-4

**Published:** 2021-09-20

**Authors:** Stephanie M. Wilson, Adam P. Maes, Carl J. Yeoman, Seth T. Walk, Mary P. Miles

**Affiliations:** 1 Department of Health and Human Development, Montana State University, Box 173540, 20 Herrick Hall, MT 59717 Bozeman, USA; 2 School of Public and Community Health Sciences, University of Montana, Missoula, USA; 3 Department of Animal and Range Sciences, Montana State University, Bozeman, USA; 4 Department of Microbiology and Cell Biology, Montana State University, Bozeman, USA

**Keywords:** Postprandial lipemia, High-fat meal, Obesity, metabolic syndrome

## Abstract

**Background:**

Dyslipidemia is a feature of impaired metabolic health in conjunction with impaired glucose metabolism and central obesity. However, the contribution of factors to postprandial lipemia in healthy but metabolically at-risk adults is not well understood. We investigated the collective contribution of several physiologic and lifestyle factors to postprandial triglyceride (TG) response to a high-fat meal in healthy, overweight and obese adults.

**Methods:**

Overweight and obese adults (n = 35) underwent a high-fat meal challenge with blood sampled at fasting and hourly in the 4-hour postprandial period after a breakfast containing 50 g fat. Incremental area under the curve (iAUC) and postprandial magnitude for TG were calculated and data analyzed using a linear model with physiologic and lifestyle characteristics as explanatory variables. Model reduction was used to assess which explanatory variables contributed most to the postprandial TG response.

**Results:**

TG responses to a high-fat meal were variable between individuals, with approximately 57 % of participants exceeded the nonfasting threshold for hypertriglyceridemia. Visceral adiposity was the strongest predictor of TG iAUC (β = 0.53, *p* = 0.01), followed by aerobic exercise frequency (β = 0.31, *p* = 0.05), insulin resistance based on HOMA-IR (β = 0.30, *p* = 0.04), and relative exercise intensity at which substrate utilization crossover occurred (β = 0.05, *p* = 0.04). For postprandial TG magnitude, visceral adiposity was a strong predictor (β = 0.43, *p* < 0.001) followed by aerobic exercise frequency (β = 0.23, *p* = 0.01), and exercise intensity for substrate utilization crossover (β = 0.53, *p* = 0.01).

**Conclusions:**

Postprandial TG responses to a high-fat meal was partially explained by several physiologic and lifestyle characteristics, including visceral adiposity, insulin resistance, aerobic exercise frequency, and relative substrate utilization crossover during exercise.

**Trial Registration:**

ClinicalTrials.gov, NCT04128839, Registered 16 October 2019 – Retrospectively registered.

**Supplementary Information:**

The online version contains supplementary material available at 10.1186/s12944-021-01543-4.

## Introduction

Obesity is a well-established risk factor for chronic disease [1] with increased adiposity, especially central obesity, present early in the impaired metabolic cascade. Increased fat deposition promotes insulin resistance, dyslipidemia [2–4], and the development of chronic conditions such as diabetes, non-alcoholic fatty liver disease, and coronary artery disease [5]. Lipids and lipoproteins play an essential role in the body, but elevated postprandial lipids are atherogenic [6]. The contribution of postprandial hyperlipidemia to atherosclerosis development is likely through a co-occurrence of endothelial dysfunction, oxidative stress, and inflammation promoted from a single feeding [7]. Epidemiological studies used fasting clinical lipid profiles to predict chronic disease prior to 2009 [8]. As individuals spend the majority of waking hours in a postprandial state, fasting lipid profiles are not reflective of the dynamic nature of lipid metabolism. Evidence now suggests that the postprandial concentration is more predictive of cardiovascular events and disease risk than fasting levels [9–12]. Postprandial triglyceride (TG) in particular has been shown to predict cardiovascular disease, myocardial infarction, ischemic heart disease, and death [13, 14].

Postprandial TG responses to a meal enriched in dietary fat show considerable interindividual variability [15] with high fat meals shown to increase circulating TG at least 50 % from fasting concentrations [4]. Postprandial hypertriglyceridemia is considered present if individuals have TG concentrations greater than 175 mg/dL or 1.98 mmol/L [16]. Prominent epidemiological studies after 2009 measured blood lipids at a single postprandial timepoint which, while more feasible for large cohorts, oversimplifies the postprandial response [11, 14] and lacks the enhanced disease prediction capacity [8]. Summary measures such as total area under the curve and incremental area under the curve (iAUC) are effective summary tools for sampling in the postprandial period. Specific to the TG response after a meal, iAUC better represents TG responses while total AUC more strongly associates with fasting TG concentrations [17]. Furthermore, iAUC serves to normalize baseline interindividual variability which allows for improved assessment of the postprandial TG-rich lipoproteins [17, 18].

Postprandial TG responses are dependent on the amount of dietary fat absorbed and packaged as chylomicrons by the intestine, hepatic clearance triacylglycerol-rich lipoproteins (TRLs), and hepatic production of very low-density lipoprotein (VLDL) [19]. These three aspects of postprandial lipid metabolism are influenced by physiologic and lifestyle factors such as age, central obesity, smoking, alcohol consumption, blood pressure, diet, gender, insulin resistance, and physical activity [3, 19–21]. Central obesity, insulin resistance, and age have been observed to increase the risk of postprandial lipemia while physical activity is one of the few factors observed to attenuate postprandial TG responses [20, 22]. Bouts of physical activity acutely increase lipoprotein lipase (LPL) activity allowing for the peripheral uptake of fatty acids for fat oxidation [23]. These factors and postprandial lipemia are strongly correlated, with regular physical activity leading to a higher oxidation of dietary fat in the postprandial period than with sedentary behavior [22, 24]. The collective impact of multiple physiologic and lifestyle factors in relation to postprandial TG responses can be difficult to ascertain across study methodologies and populations and warrants further investigation.

A better understanding of postprandial TG determinants may increase disease prediction capabilities and allow for more targeted clinical strategies to improve lipid profiles and lower downstream disease risk. In this study, we investigated the postprandial lipemic response to a single high-fat meal in healthy, nondiabetic overweight and obese adults and assessed several factors known to influence the postprandial TG response [20]. We hypothesized that examination of these factors in such a metabolically at-risk cohort would identify early processes of metabolic dysregulation involved in postprandial lipemia.

## Methods

### Ethics Statement

The protocol was approved by the Institutional Review Board at Montana State University and followed the guidelines of the Declaration of Helsinki. Written informed consent was obtained from all participants prior to their participation. The original study was retrospectively registered October 2019 at ClinicalTrials.gov (NCT04128839).

### Study Population

Potential participants were recruited via advertisement between March 2016 to June 2018 for a study which assessed the inflammation-lowering impact of Aronia berries in a humanized mouse model. Our specific analysis focuses on the human cohort recruited for this study and thus, a secondary endpoint of the original study. Inclusion criteria included being between 18 and 55 years old and having a BMI between 27 and 36 kg/m^2^. Criteria for exclusion included antibiotics within 90 days of study enrollment, regular use of anti-inflammatory medications, use of estrogen-only contraceptives, wheat and/or dairy allergies or intolerances, were pregnant, or had any musculoskeletal, cardiovascular, gastrointestinal, or immunological condition that could interfere with the study. All potentially eligible participants were screened over the phone for inclusion and exclusion criteria.

### Research Design

The study followed a cross-sectional design. Participants were asked to attend two visits in the Nutrition Research Laboratory at Montana State University. The initial visit involved questionnaires and analysis of body composition and cardiorespiratory fitness. The second visit occurred within two weeks after the first visit and involved blood collection before and after a high-fat meal challenge. Fasting and postprandial lipids, glucose, and insulin were measured for four hours postprandially. Blood pressure, visceral adipose tissue, physical activity frequency, and substrate utilization crossover during a submaximal exercise test were measured.

### Physical Activity Frequency

Participants were asked to complete a written 3-question questionnaire on their physical activity in the past week. Questions were taken from the FITNESSGRAM Test Administration Manual and in brief, asked “On how many of the past 7 days did you -” perform 30–60 min of aerobic exercise, strengthening activities, and stretching exercise with written examples of each provided for reference [25].

### Anthropometrics

Measurements were collected from participants using the validated segmental multifrequency bioelectrical impedance analysis (SECA mBCA 515, Hamburg, Germany) [26]. Participants were instructed to refrain from eating, drinking, or exercising in the three hours prior to testing. Fat mass percentage and estimated visceral adipose in liters were used for analysis.

### Blood Pressure

Systolic and diastolic blood pressure measurements were performed on seated participants after 5–10 min of rest. Two automated measurements were taken with the average of the measurements used for analysis.

### Cardiorespiratory Fitness

Participants were asked to complete a modified Bruce protocol on a treadmill for determination of calculated absolute oxygen consumption (VO_2_) max at their age-predicted heart rate max. Speed and grade of the treadmill (Woodway GmbH D-79,576, Weil am Rhein, Denmark) were manually changed by the researcher with each progressive three-minute stage until the participant reached 85 % of their age-predicted maximal heart rate. Expired gases were collected and averaged every 15 s for analysis through a metabolic cart system (ParvoMedics, TrueMax 2400 Metabolic System, Sandy, Utah, USA). Heart rate (bpm) and VO_2_ (ml/kg/min) data from each participant were input into a simple linear regression model to predict the absolute VO_2_ at the age-predicted maximal heart rate based on the equation presented by Tanaka, Monahan, and Seals [27].

Exercise requires metabolically flexibility, the ability to switch between glucose and fat use in response to metabolic demand [28]. Through standard stoichiometric equations and indirect calorimetry methods like the metabolic cart system, utilization of fats and lipids are able to be quantified as kcal during the exercise protocol. The switch or “crossover” point of substrate utilization during exercise reflects the point at which kilocalories per minute of carbohydrate expended exceeded that from fat. The crossover point was determined as the point at which carbohydrate and fat expenditure most rapidly and proportionately differentiated from the other [29]. More information about how the crossover percentage was calculated is available in Additional File 1. Using the VO_2_ at the crossover point, we derived the crossover percentage of the calculated absolute VO_2_ max.

### High-Fat Meal Challenge

The high-fat meal challenge was performed after an overnight fast (10–12 h) during the morning hours. The meal consisted of three pieces of toasted whole wheat bread (Wheat Montana) and approximately 58.3 g of salted butter (Tillamook). Total energy content of the high-fat meal challenge was 714 kcal with a macronutrient breakdown of 50 g fat, 54 g carbohydrate, and 12 g protein. Approximately 43.1 % of the caloric content was from fat and saturated fats making up approximately 57 % of the total fat load. The meal contained approximately 9 g of dietary fiber. Water was provided with the meal and caffeinated early grey black tea (Bigelow) was provided for participants who identified as habitual coffee consumers. Participants were asked to consume the meal in 15 min, and the postprandial period timing began when participants started the meal.

### Blood Sampling

Participants were instructed to avoid alcohol consumption and strenuous physical activity in the 24 h before blood collection and to complete an overnight fast (10–12 h) before blood collection. Venous blood samples were collected through a cannula inserted into the antecubital vein after a 3-mL waste withdrawal, then followed by a sterile saline flush performed by a physician or nurse on the research team. The fasting sample was drawn 30 min after catheter insertion. After meal ingestion, blood was drawn every hour for four hours in the postprandial period, totaling five time points including fasting. This frequency of blood sampling has been previously shown in healthy populations to accurately describe postprandial lipemia to a high fat meal and are also predictive of 8-hour responses [30, 31]. Blood was collected into 8.5 mL endotoxin-free serum separating and 4.0 mL heparinized vacutainer tubes (BD Vacutainer, Franklin Lakes, New Jersey, USA). The serum tube was allowed to clot for 15 min at room temperature before centrifugation (3000 rpm, 15 min). Serum aliquots were frozen at -80ºC until analysis.

### Biochemical Analyses

Blood triglycerides, glucose, and high-density lipoprotein were determined using the Picollo Xpress Chemistry Analyzer lipid panels (Abaxis, Union City, USA). Insulin was determined through ELISA (MP Biomedicals, USA) performed according to manufacturer instructions, with the average used for analysis. Mean inter-assay coefficient of variation for samples run in duplicate was 13.3 %.

### Insulin Resistance

Fasting blood glucose and insulin were used to determine the homeostatic model of insulin resistance (HOMA-IR) in the original HOMA-IR formula [32]:

$$\frac{Glucose \left(\frac{mmol}{L}\right)*Insulin\left(\frac{mU}{L}\right)}{22.5}$$

### Postprandial Lipemic Response

The postprandial lipemic response to the high-fat meal was summarized as iAUC, a calculation method that accurately represents the postprandial TG response to a high-fat meal [17]. The magnitude of the postprandial lipemic response was also calculated by subtracting the fasting TG value from the maximum TG value during the 4-hr postprandial period after the high-fat meal.

### Statistical Analysis

Analysis was conducted in RStudio (1.3.1073) running R 4.0.2 [33], and data was visualized using *ggplot2* [34].

To assess which variables most influence TG iAUC and the TG magnitude, initial saturated multivariate linear regression models were created with the following predictor variables: age, sex, relative exercise intensity of substrate utilization crossover, visceral adipose tissue in liters, HOMA-IR, systolic and diastolic blood pressure in mmHg, and aerobic exercise frequency. Model refinement was performed by stepping down one main effect at a time from the initial model. Model reduction was determined through strength of evidence against the null hypothesis using Type III F-tests, in which every test is conditional on every variable in the model. Model refinement stopped when the majority of predictor variables reached their smallest p-value. Final linear models were screened for shared information among predictor variables using variance inflation factors from the *car* package [35], with values > 5 set as the threshold for predictor removal. Further validity conditions were confirmed through residual visualizations. Power was calculated *a posteriori* from the final TG iAUC regression model using the pwr.f2.test in the *pwr* package. Computed power was 95 % at a Type I probability of 0.05.

## Results

**Fig. 1:**
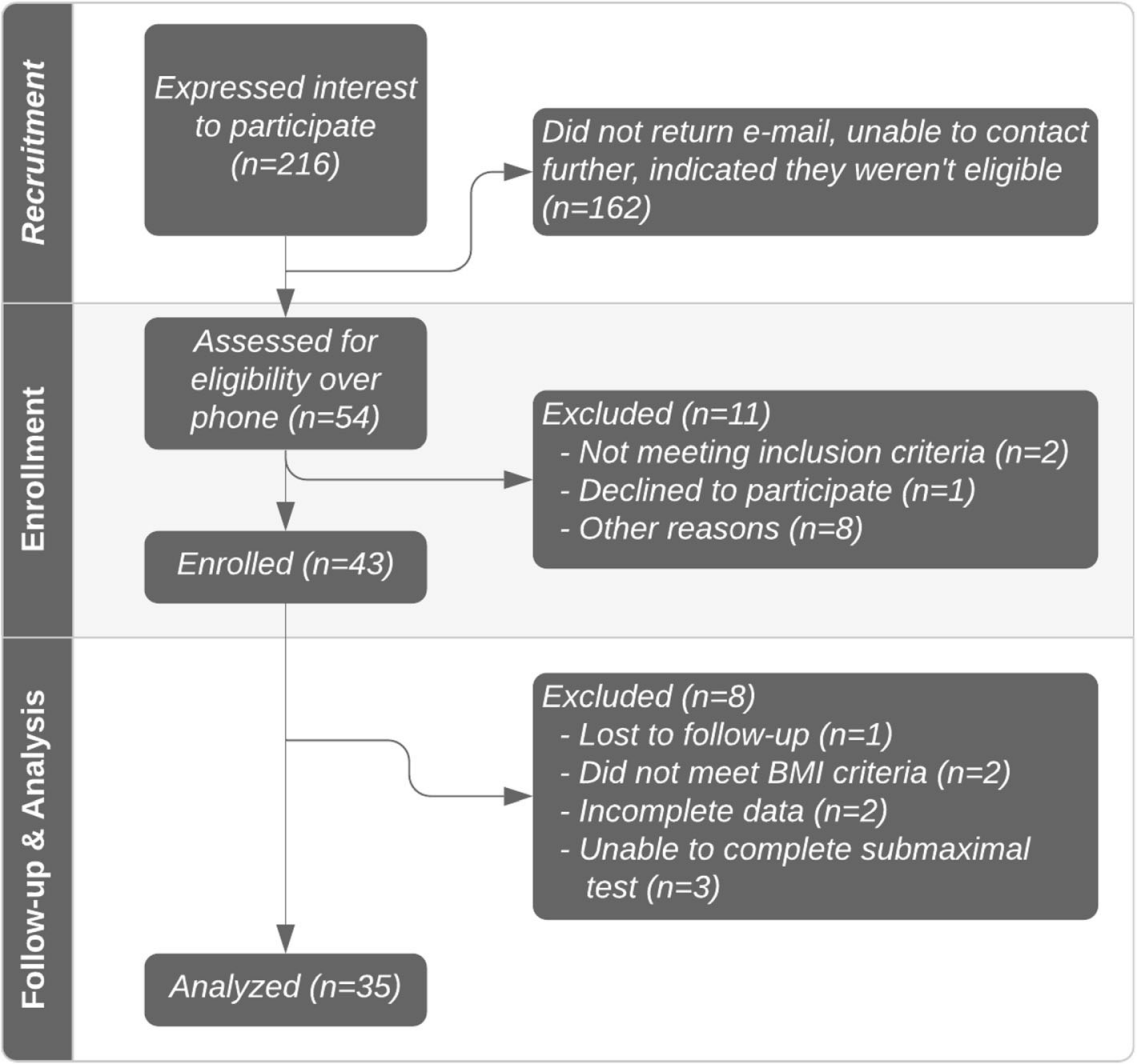
A flowchart of the study design. Flyers and e-mails were used to recruit interested individuals. Interested individuals contacted the research team, who sent additional information about the study. Fifty-four individuals completed a phone screening with the same researcher with those individuals who met eligibility requirements, enrolled to participate. Forty-three individuals enrolled. Of these, two individuals were found to be ineligible at the first study visit and one individual was unable to be contacted thus leaving forty individuals who completed the high-fat meal challenge at the second study visit

Of the initial 216 individuals contacted, we assessed the eligibility of 54 individuals through a screening phone call. Forty-three individuals consented to participate in the study (Fig. 1). Two individuals were confirmed to be ineligible based on confirmation of BMI criteria at study onset, and one individual was unable to be reached to complete the full study. Thus, 40 overweight and obese men and women (body mass index, BMI 27–36 kg/m^2^) between the ages of 18 and 55 participated in testing of cardiovascular, anthropometric, and metabolic markers and ingestion of a 50 g high-fat meal challenge. Five participants were excluded from analysis due to incomplete data (n = 2) or inability to complete the submaximal treadmill test (n = 3). On average, participants were obese according to BMI and had slightly elevated fasting triglycerides but were otherwise within normal ranges for other metabolic syndrome criteria (Table 1), according to the National Cholesterol Education Program Adult Treatment Panel III definition [36]. Approximately 74 % of our cohort did not have metabolic syndrome (Additional File 2).

**Table 1:** Participant characteristics (*n* = 35)

	**Mean (SD**)
Men/Women	16/19
Age (years)	36.6 (10.2)
BMI (kg/m^2^)	30.4 (1.8)
Fat Mass (%)	34.9 (6.6)
Waist Circumference (cm)	95.7 (9.6)
VAT (l)	2.2 (1.4)
VO_2_(ml/kg/min)	44.1 (8.6)
HbA1c (%)	5.3 (0.3)
Fasting GLU (mmol/L)	5.4 (0.4)
Fasting TG (mmol/L)	1.7 (1.1)
Fasting CHOL (mmol/L)	4.7 (0.9)
Fasting HDL (mmol/L)	1.3 (0.4)
Systolic BP (mmHg)	114 (13.5)
Diastolic BP (mmHg)	76 (9.5)

Abbreviations: body mass index, *BMI*; visceral adipose tissue, *VAT*; maximal oxygen consumption, *VO*_2_; hemoglobin *A1C*, HbA1c; glucose, *GLU*; triglycerides, *TG*; cholesterol, *CHOL*; high-density lipoprotein, *HDL*; systolic blood pressure, *SBP*; diastolic blood pressure, *DBP*.

### Postprandial Lipemic Response to the High-fat Meal

**Fig. 2:**
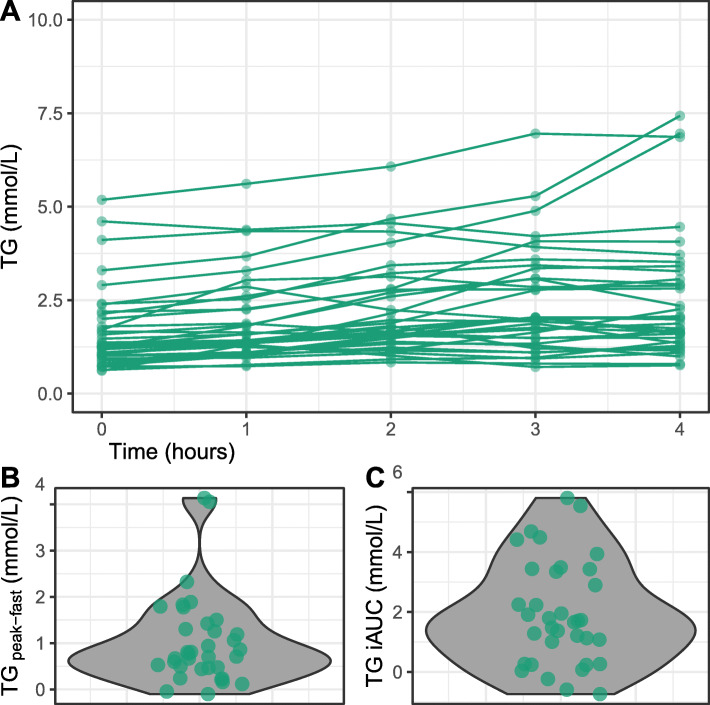
Overview of postprandial responses to a high-fat meal challenge. (**A**) Individual postprandial blood TG responses to the high-fat meal challenge, (**B**) Violin plot of the postprandial TG magnitude distribution and individual values, and (**C**) Violin plot of TG iAUC distribution and individual values. Responses throughout the high-fat meal challenge showed high individual variability. Acronyms: TG, triglycerides; iAUC, incremental area under the curve

Participants entered the high-fat meal challenge with fasting TG concentrations from normal (< 1.7 mmol/L) to moderately elevated (2.0–5.6 mmol/L) levels but displayed high interindividual variability in blood TG concentrations during the 4-hr postprandial period (Fig. 2 A). During the high-fat meal challenge, 25 participants had peak TG values above the threshold of 1.98 mmol/L (175 mg/dL) for hypertriglyceridemia diagnosis in nonfasting states [16]. The TG response of 35 participants to the high-fat meal challenge was summarized as iAUC and as postprandial magnitude change from fasting concentrations. Most participants had a small-to-modest rise in TG with a median (mean) increase of + 0.77 mmol/L (1.04 mmol/L), but two participants had a TG increase greater than 4 mmol/L (Fig. 2 B). The median (mean) TG iAUC was + 1.70 mmol/L (1.96 mmol/L) and overall, a normalization of interindividual variability was observed (Fig. 2 C).

To identify factors that predicted TG iAUC and TG magnitude, model refinement was performed from a saturated multivariate linear model that included factors previously shown to influence postprandial lipid metabolism (Additional File 3, Additional File 4). A summary of the final models can be found in Additional File 5, and the full estimated regression equations can be found in Additional File 6. Both models shared the same predictor variables, with the model for postprandial TG magnitude additionally including systolic blood pressure. The predictor variables explain approximately 28.6 % of the variance in TG iAUC and approximately 30.4 % in TG magnitude according to the adjusted R^2^.

**Fig. 3:**
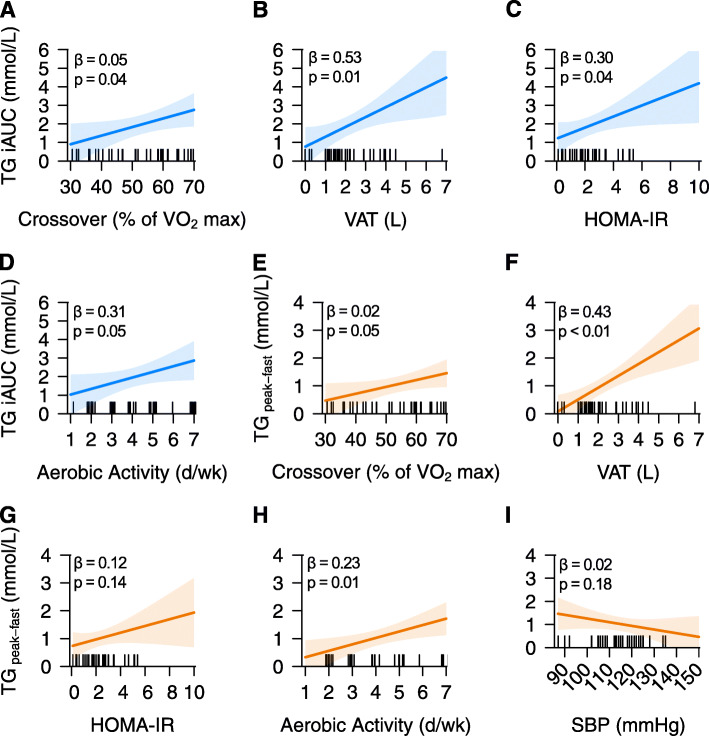
Estimated beta coefficients for TG iAUC (**A**-**D**) and the postprandial TG magnitude (**E**–**I**) models. Plots for TG iAUC based on substrate utilization crossover during a submaximal exercise test as a percentage of estimated VO_2_ max (**A**), visceral adipose (**B**), HOMAIR (**C**), and aerobic exercise frequency in the last 7 days (**D**). Plots for postprandial TG magnitude based on substrate utilization crossover as a percentage of estimated VO_2_ max (**E**), VAT (**F**), HOMAIR (**G**), aerobic exercise frequency in the last 7 days (**H**), and systolic blood pressure (**I**). Bands indicate 95% confidence intervals and ticks on the x-axis indicate observed data from participants. *P*-values for effects are available in Additional File 3 and Additional File 4. Acronyms: VAT, visceral adipose tissue; HOMA-IR, homeostatic model of insulin resistance; SBP, systolic blood pressure; TG, triglycerides; iAUC, incremental area under the curve.

Visceral adiposity was the best predictor of TG iAUC followed in unit change by HOMA-IR, aerobic exercise frequency, and relative exercise intensity of substrate utilization crossover. A modest increase was seen with a later shift to carbohydrate metabolism during a submaximal exercise test (Fig. 3 A). Increased visceral adiposity (Fig. 3 B), HOMA-IR (Fig. 3 C), and aerobic exercise frequency (Fig. 3 D) were associated with greater increases in TG iAUC.

Postprandial TG magnitude was also strongly predicted by visceral adiposity. Aerobic exercise and relative exercise intensity of substrate utilization crossover were also determined predictors. As observed with TG iAUC, participants had a modest increase in postprandial TG magnitude with the relative exercise intensity of substrate utilization crossover (Fig. 3 E) and greater changes in postprandial TG magnitude with increased visceral adiposity (Fig. 3 F). Changes in HOMA-IR were associated with a slight increase in magnitude; however, little evidence as indicated by the poor strength of evidence supports this as a predictor (Fig. 3 G). Aerobic exercise frequency was additionally a modest predictor of magnitude (Fig. 3 H). Lastly, systolic blood pressure was the sole predictor not shared between the two models. While systolic blood pressure was the only negative association in the model, there was little evidence to support it as a predictor of postprandial TG magnitude (Fig. 3 I).

## Discussion

In the present study, we investigated the acute postprandial effect of a single high-fat meal challenge on blood triglyceride levels in healthy overweight and obese adults. Our meal with butter fat was rich in saturated fatty acids that have been shown to elicit greater postprandial TG responses than meals with mono- or polyunsaturated fatty acids [37]. By analyzing hourly over a 4-hour postprandial period, we were able to quantify the TG response in two succinct ways, (1) iAUC and (2) the magnitude of the postprandial response from relative to fasting TG concentrations. In addition, we were able to determine which biological and lifestyle factors held the greatest predicative power in TG response prediction, especially VAT, insulin resistance, aerobic exercise frequency, and relative exercise intensity of substrate utilization crossover.

On average, our overweight and obese participants had slightly elevated fasting TG but normal blood glucose, blood pressure, waist circumference and high-density lipoprotein (HDL). This finding was unsurprising as approximately 31 % of US adults have elevated fasting TGs above approximately 1.70 mmol/L (150 mg/dL) with increased fasting lipids positively correlated to BMI [4]. Despite participants overall classifying as metabolically healthy, 57 % of our participants had peak postprandial TG above the diagnostic threshold for nonfasting triglyceridemia of 1.98 mmol/L (175 mg/dL), as determined from the Women’s Health Study where a single nonfasting time point was used to analyze risk of cardiovascular disease events [16]. In the analysis of the Women’s Health Study, cardiovascular event risk was greater (Hazard Ratio, 2.05) with nonfasting TG above 1.98 mmol/L in the 0–4 h since the last meal. The amount of fat present in their meals was not reported, thus limiting direct comparison to the present study. Large cohort studies have found maximal TG changes after normal food intake ranging from + 0.1 to 0.3 mmol/L (+ 8.86 to 26.57 mg/dL) [8], which is notably lower than the mean 1.0 mmol/L (88.57 mg/dL) magnitude we observed in the 4-hour postprandial sampling period after the 50 g high-fat meal. As previously observed in the PREDICT I study, we also observed high interindividual variability in postprandial TG responses which supports use of postprandial response summary measures like iAUC [15].

Of the eight physiologic and lifestyle factors included in our models, visceral adiposity was the strongest predictor of TG iAUC and magnitude following the high-fat meal challenge. Central adiposity, more so than peripheral subcutaneous fat, is strongly linked to numerous metabolic abnormalities such as insulin resistance [38, 39] and chronic inflammation as well as to fasting hypertriglyceridemia [40, 41]. Our finding supports previous work with healthy adults with varying levels of body fat which found a positive correlation between the TG response and visceral adipose tissue [42]. The impact of visceral adiposity superseded the sex effect in both our statistical models, a finding also observed previously [42] and is notable in that gender differences have been found in adipose LPL activity [43]. Adipose tissue is an active endocrine organ that influences glucose and lipid metabolism through various adipokines [38, 44], with visceral adipocytes in particular exhibiting greater lipolytic activity through LPL [44]. Insufficient LPL activity has been previously suggested as an early factor in atherosclerosis [6]. Thus, lifestyle strategies to reduce visceral adipose tissue may have implications for improved postprandial TG response in addition to overall metabolic health.

While hypertriglyceridemia stemming from VAT is generally attributed, at least in part, to insulin resistance, our findings indicate that insulin resistance more closely associated with a sustained elevation in TG than the peak magnitude of the TG response. Participants with a greater degree of insulin resistance displayed a higher postprandial TG response than individuals who were more insulin sensitive, a finding previously observed in studies with healthy and obese individuals [45, 46]. Postprandially, insulin exerts an antilipolytic effect through hormone sensitive lipase, promotes triacylglycerol synthesis, and activates LPL in adipose tissue which is responsible for clearance of triacylglycerol from plasma [47]. In an insulin resistant state, free fatty acids increase as lipolysis is not suppressed and de novo lipogenesis occurs from hyperglycemia which helps in part to explain the sustained elevated TG observed in our study [48]. In turn, the liver secretes more TG-rich VLDL and may exhibit fat accumulation, pushing a feedforward loop advancing insulin resistance. Insulin resistance also promotes increased chylomicron secretion from the intestine and the general postprandial accumulation of TRLs from the liver and intestine [49–51]. Improved insulin sensitivity through physical activity may not only improve glucose transport [52] but may additionally prevent the accumulation of TRLs and reduce the overall TG response in the postprandial period.

Contrary to our expectation that greater frequency of aerobic activity, higher cardiorespiratory fitness, and substrate crossover from fat to carbohydrate at a higher relative exercise intensity would be inversely related to postprandial TG magnitude and TG iAUC, we measured a positive relationship with frequency of aerobic activity and crossover intensity and no relationship with cardiorespiratory fitness. This is a notable finding as habitual physical activity has not been found to be a key determinant of postprandial TG responses [3]. Chronic exercise improves the overall fasting lipid profile through modulation of apolipoprotein A1 expression and increase in HDL by way of increased lecithin-cholesterol acyltransferase activity [53]. Acute exercise has been found to lower the magnitude of the postprandial lipemic response through decreased chylomicron appearance [23], increased oxidation of fatty acids post-exercise [54], decreased VLDL secretion from the liver, and increased LPL activity [23] though LPL activity in particular may be enhanced in men compared to women post-exercise [55]. An energy deficit post-exercise has been suggested as the primary factor in the attenuation of postprandial TG response [56]. Given the positive association of postprandial TG magnitude and TG iAUC with VAT and insulin resistance and no association with cardiorespiratory fitness, it is possible that the self-reported frequency of aerobic activity reflects perception of effort during activities that do not correspond to beneficial impacts on cardiorespiratory fitness. In light of the limited number of studies reporting the relationship between physical activity and postprandial TG, this a notable finding but one that requires further exploration.

The relationship between physical activity and postprandial TG magnitude and TG iAUC was further explored by evaluating relative exercise intensity at which substrate utilization crossover occurred and TG iAUC to the high-fat meal. The percent of VO_2_ max at which the rise in carbohydrate oxidation matched fat oxidation and crossed over to begin dominating as fuel was also a predictor of both TG iAUC and postprandial TG magnitude, but one that impacted the response less than visceral adiposity and aerobic exercise frequency. After normalization to estimated VO_2_ max, overweight and obese adults with a later shift to carbohydrate utilization during exercise had greater TG iAUC and postprandial TG magnitude to a high-fat meal challenge. To the authors knowledge, this is the first study to examine substrate utilization crossover during submaximal exercise in comparison to postprandial TG responses. Exercise requires an increase in energy expenditure and warrants a shift in energy usage from lipids at low-to-moderate intensity to carbohydrate at high intensity exercise [57]. The inability to effectively and rapidly move between energy systems in response to changing energy requirements and demands is considered metabolic inflexibility [58]. An earlier shift to carbohydrate oxidation during exercise has been noted in sedentary overweight versus normal weight controls and while the exact mechanisms for reduced fat oxidation are unknown, it is generally attributed to impaired muscle substrate utilization [59]. Submaximal exercise tests can serve as a way to assess the operation status of the body’s oxidative machinery, allowing us to detect fat oxidation impairments that precede insulin resistance and possibly influence lipid handling during high lipid availability.

Collectively, increased postprandial TG magnitude and TG iAUC associated positively with VAT, insulin resistance, and relative exercise frequency for substrate utilization crossover while associating negatively with reported frequency of aerobic activity may be a serendipitous finding relevant to exercise training response heterogeneity. This apparent paradox is consistent with the influence of having a high risk for diabetes stemming from familial history or history of gestational diabetes. In one study, relatives of individuals with type 2 diabetes had lower insulin sensitivity and those who did not respond to exercise training with increased insulin sensitivity also did not improve ATP production capacity and increased intrahepatic and intramuscular lipid concentrations [60, 61]. Increased uptake of fatty acids into tissues in individuals with greater VAT and insulin resistance could attenuate postprandial TG responses owing to greater removal of fatty acids from TG in postprandial chylomicrons. Increased abundance of the FAT/CD36 plasma membrane fatty acid transport system is well documented in rats with type 2 diabetes [62, 63]. The possibility of coupling postprandial TG responses with a variable such as substrate crossover point to increase sensitivity of diabetes risk prediction is exciting and worthy of further exploration.

Strengths of the present study include fasting and hourly postprandial lipid measurements in a group of healthy overweight and obese adults with varying levels of body fat percentage and cardiovascular fitness. Our statistical model predictors explain 28–30 % of the variance in the TG iAUC and TG postprandial magnitude. However, our findings are limited to this study meal as varying meal components including fiber and even dairy-based meals matched for fat content affect postprandial responses differently [19, 64]. Additionally, while participants were asked to avoid strenuous exercise and alcohol before blood collection, it is possible they exercised or consumed alcohol the day prior which alter postprandial TG responses. We also recognize factors may be in play that were not measured in the current study. While participants were asked to perform an overnight fast proceeding blood collection, we did not control the fat to carbohydrate ratio in the evening meal which may have influenced the glucose and lipid metabolism the following morning [65]. Furthermore, the inclusion of diet history questionnaires to assess habitual dietary patterns may have potentially informed which metabolic pathways in postprandial lipemia are altered [19]. As we observed a positive relationship between aerobic exercise frequency and TG iAUC, further research in high-fat meal induced postprandial lipemia may benefit by quantifying the frequency, intensity, and duration of physical activity through objective and subjective methods.

In conclusion, our findings support that several physiologic and lifestyle factors including central adipose accumulation, insulin resistance, and exercise are collectively implicated in the metabolic regulation of postprandial lipemia and can be used to predict postprandial TG responses to a high-fat meal. These factors are interrelated and subsequently, must be jointly taken into account in strategies to reduce postprandial lipemia in healthy but at-risk populations. By using appropriate postprandial TG summary measures, we found that healthy, nondiabetic overweight and obese adults with increased visceral adipose tissue and insulin resistance had, on average, greater postprandial TG iAUC to a high fat meal test. We also found that aerobic exercise frequency and the increased ability to use fat during an aerobic exercise test was positively correlated to postprandial TG responses. The present study also highlights the potential value of measuring postprandial TG responses using a standardized challenge for prediction of type 2 diabetes risk and the need to further research in this area.

## Supplementary Information

**Additional File 1: **Determination of the percentage of VO_2_ max at substrate utilization crossover point. Visual provides additional detail using real participant data on how crossover percentage was determined.

**Additional File 2: **Presence of metabolic syndrome criteria in analyzed study cohort (n = 35). Criteria were based of the National Cholesterol Education Program Adult Treatment Panel III definition of metabolic syndrome [32]. This metabolic syndrome definition includes criteria on central obesity, hypertension, insulin resistance, and dyslipidemia.

**Additional File 3: ***P*-values decisions during model refinement for the TG iAUC Response. Bolded items indicate the variable that was dropped at each decision (D) step. The final model included variables from D4, with D5 shown to confirm D4 was the final model. No further predictor variables were removed according to variance inflation factor values.

**Additional File 4: ***P*-values decisions during model refinement for the TG change (peak – fasting) Response. Bolded items indicate the variable that was dropped in the decision (D) step. The final model included the variables from D3, with D4 shown to confirm D3 was the final model. No further predictor variables were removed according to variance inflation factor values.

**Additional File 5: **Model summaries for TG iAUC response and postprandial TG magnitude. The table is a complement to Fig. 3 and provides additional information on predictor variables as well as F-statistics from statistical output.

**Additional File 6: **The estimated regression equations for TG iAUC response and postprandial TG magnitude after model refinement. In the equations: i is subject, crossover is the percentage of VO_2_ max where carbohydrate became the dominant substrate utilized, VAT is visceral adipose tissue in liters, HOMA-IR is insulin resistance, aerobic is self-reported days with aerobic exercise in last 7 days, SBP is systolic blood pressure in millimeters of mercury, and ε_i_ ~ N(0, σ^2^_ε_).

## Data Availability

Participant data is not publicly available due to them containing information that could compromise research participant privacy, but the minimal data are available from the corresponding author on reasonable request.

## Abbreviations

BMI: Body mass index
HDL: High density lipoprotein
HOMA-IR: Homeostatic model of insulin resistance
iAUC: Incremental area under the curve
LPL: Lipoprotein lipase
TG: Triglyceride
TRLs: Triacylglycerol rich lipoproteins
VLDL: Very low-density lipoprotein
VO_2_: Oxygen consumption
