# Supplementary material for: Determinants of the postprandial triglyceride response to a high-fat meal in healthy overweight and obese adults
Source: Lipids Health Dis. 2021 Sep 20;20:107. doi: 10.1186/s12944-021-01543-4 (PMC8451105; doi:10.1186/s12944-021-01543-4)
Supplement: Supplementary file 1 — Additional File 1: Determination of the percentage of VO2 max at substrate utilization crossover point. Visual provides additional detail using real participant data on how crossover percentage was determined. [file 12944_2021_1543_MOESM1_ESM.pdf]

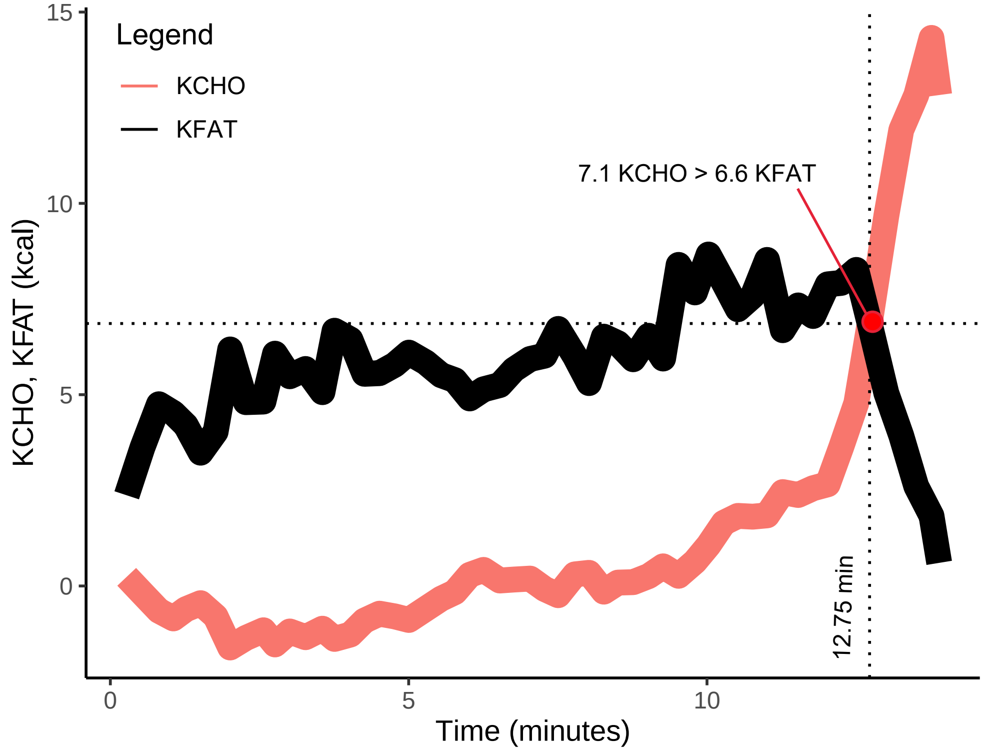

Example determination of Crossover Percentage
Data in this example is from one female participant.

Time at which KCHO>KFAT = 12.75 minutes
VO_2/kg at 12.75 minutes = 35.54 ml/kg/min

Crossover VO_2 / Calculated absolute VO_2 max = % of VO_2 max at crossover (ie, Crossover %)
35.54 ml/kg/min / 50.72 ml/kg/min = **68.1%**
