# Supplementary figures and images for: Determinants of the postprandial triglyceride response to a high-fat meal in healthy overweight and obese adults
Source: Lipids Health Dis. 2021 Sep 20;20:107. doi: 10.1186/s12944-021-01543-4 (PMC8451105; doi:10.1186/s12944-021-01543-4)

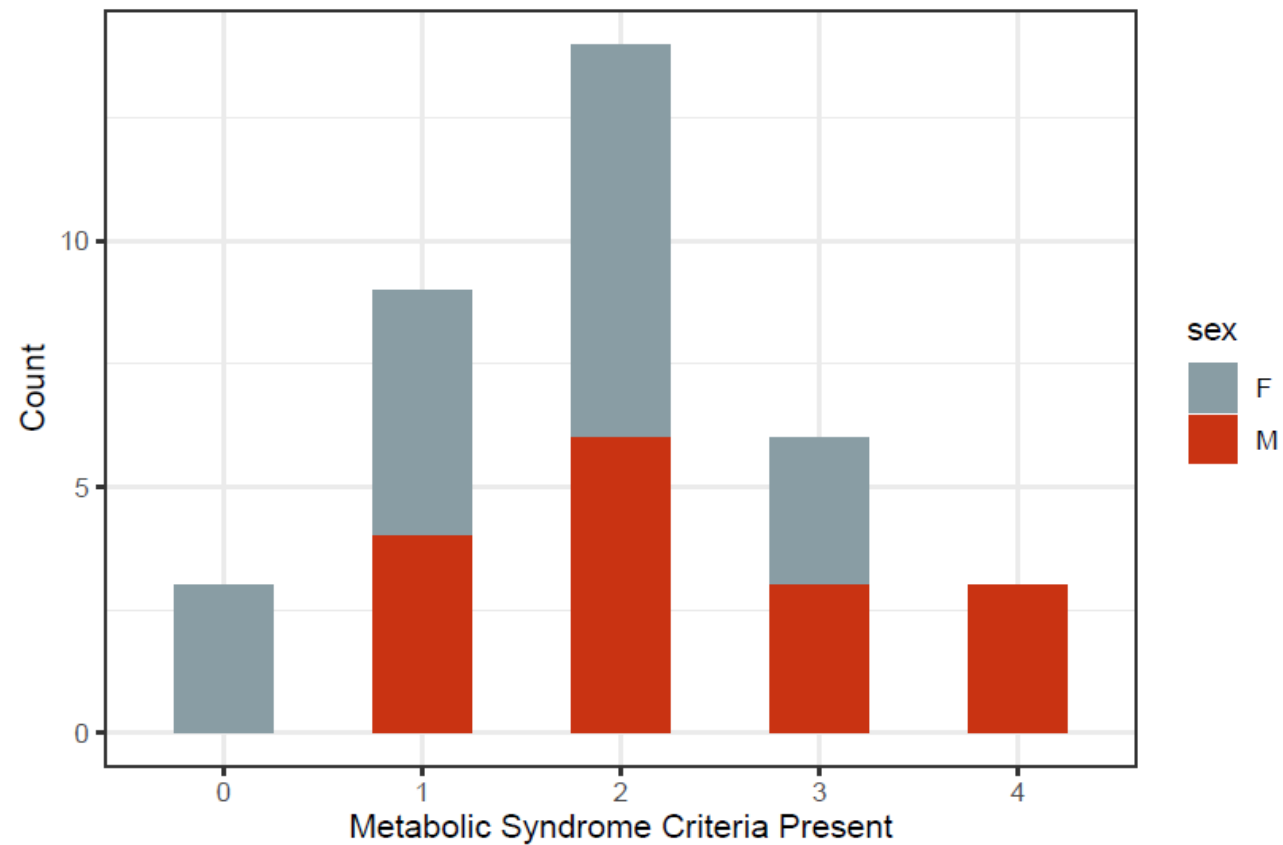

Supplement: Supplementary file 2 — Additional File 2: Presence of metabolic syndrome criteria in analyzed study cohort (n = 35). Criteria were based of the National Cholesterol Education Program Adult Treatment Panel III definition of metabolic syndrome [32]. This metabolic syndrome definition includes criteria on central obesity, hypertension, insulin resistance, and dyslipidemia. [file 12944_2021_1543_MOESM2_ESM.pdf]
