# Supplementary material for: Determinants of the postprandial triglyceride response to a high-fat meal in healthy overweight and obese adults
Source: Lipids Health Dis. 2021 Sep 20;20:107. doi: 10.1186/s12944-021-01543-4 (PMC8451105; doi:10.1186/s12944-021-01543-4)
Supplement: Supplementary file 3 — Additional File 3: P-values decisions during model refinement for the TG iAUC Response. Bolded items indicate the variable that was dropped at each decision (D) step. The final model included variables from D4, with D5 shown to confirm D4 was the final model. No further predictor variables were removed according to variance inflation factor values. [file 12944_2021_1543_MOESM3_ESM.pdf]

	Age	Sex	Crossover	VAT	HOMAIR	Aerobic	SBP	DBP	R^2_{adj}
Full	0.671	0.797	0.004	0.117	0.043	0.058	0.555	**0.807**	0.195
D1	0.704	**0.843**	0.042	0.101	0.041	0.052	0.474	NA	0.223
D2	**0.727**	NA	0.033	0.015	0.037	0.048	0.478	NA	0.249
D3	NA	NA	0.032	0.011	0.032	0.046	**0.518**	NA	0.272
D4*	NA	NA	0.036	0.013	0.037	**0.054**	NA	NA	0.286
D5	NA	NA	0.090	0.050	0.083	NA	NA	NA	0.217

*Final model. Abbreviations: DBP, diastolic blood pressure; HOMAIR, homeostatic model of insulin resistance; SBP, systolic blood pressure; VAT, visceral adipose tissue.
