# Supplementary material for: Determinants of the postprandial triglyceride response to a high-fat meal in healthy overweight and obese adults
Source: Lipids Health Dis. 2021 Sep 20;20:107. doi: 10.1186/s12944-021-01543-4 (PMC8451105; doi:10.1186/s12944-021-01543-4)
Supplement: Supplementary file 4 — Additional File 4: P-values decisions during model refinement for the TG change (peak – fasting) Response. Bolded items indicate the variable that was dropped in the decision (D) step. The final model included the variables from D3, with D4 shown to confirm D3 was the final model. No further predictor variables were removed according to variance inflation factor values. [file 12944_2021_1543_MOESM4_ESM.pdf]

	Age	Sex	Crossover	VAT	HOMAIR	Aerobic	SBP	DBP	R^2_{adj}
Full	**0.820**	0.524	0.046	0.052	0.150	0.019	0.546	0.809	0.242
D1	NA	0.543	0.042	0.034	0.132	0.017	0.569	**0.751**	0.269
D2	NA	**0.475**	0.037	0.032	0.123	0.011	0.139	NA	0.292
D3*	NA	NA	0.046	<0.001	0.141	0.012	**0.182**	NA	0.304
D4	NA	NA	0.075	0.002	**0.279**	0.023	NA	NA	0.283

*Final model. Abbreviations: DBP, diastolic blood pressure; HOMAIR, homeostatic model of insulin resistance; SBP, systolic blood pressure; VAT, visceral adipose tissue.
