# Supplementary material for: Determinants of the postprandial triglyceride response to a high-fat meal in healthy overweight and obese adults
Source: Lipids Health Dis. 2021 Sep 20;20:107. doi: 10.1186/s12944-021-01543-4 (PMC8451105; doi:10.1186/s12944-021-01543-4)
Supplement: Supplementary file 5 — Additional File 5: Model summaries for TG iAUC response and postprandial TG magnitude. The table is a complement to Fig. 3 and provides additional information on predictor variables as well as F-statistics from statistical output. [file 12944_2021_1543_MOESM5_ESM.pdf]

Model	Coefficient	F-statistic	p-value
$\widehat{TG}_{iAUC}$			
Crossover[1] (%)	0.05	4.80	0.04
VAT (L)	0.53	7.05	0.01
HOMA-IR	0.30	4.78	0.04
Aerobic Frequency[2] (d/wk)	0.31	4.00	0.05
$\widehat{TG}_{(peak-fast)}$			
Crossover (%)	0.02	4.36	0.05
VAT (L)	0.43	14.0	<0.01
HOMA-IR	0.12	2.30	0.14
Aerobic Frequency (d/wk)	0.23	7.15	0.01
SBP (mmHg)	0.02	1.87	0.18

[1]Crossover indicates the percentage of VO_2 max during a submaximal exercise test where carbohydrate (versus fat) became the dominant substrate utilized.

[2]Aerobic frequency indicates the self-reported number of days where participants completed aerobic exercise in last 7 days.

Abbreviations: maximal oxygen consumption, VO_2; visceral adipose tissue, VAT; homeostatic model of insulin resistance, HOMA-IR; systolic blood pressure, SBP.
