# Supplementary material for: Determinants of the postprandial triglyceride response to a high-fat meal in healthy overweight and obese adults
Source: Lipids Health Dis. 2021 Sep 20;20:107. doi: 10.1186/s12944-021-01543-4 (PMC8451105; doi:10.1186/s12944-021-01543-4)
Supplement: Supplementary file 6 — Additional File 6: The estimated regression equations for TG iAUC response and postprandial TG magnitude after model refinement. In the equations: i is subject, crossover is the percentage of VO2 max where carbohydrate became the dominant substrate utilized, VAT is visceral adipose tissue in liters, HOMA-IR is insulin resistance, aerobic is self-reported days with aerobic exercise in last 7 days, SBP is systolic blood pressure in millimeters of mercury, and εi ~ N(0, σ2ε). [file 12944_2021_1543_MOESM6_ESM.pdf]

$$\widehat{TG}_{iAUC,i} = -3.67 + 0.05*X_{Crossover,\,i} + 0.53*X_{VAT,\,i} + 0.30*X_{HOMAIR,\,i} + 0.31*X_{Aerobic,\,i} + \varepsilon_i$$

$$\widehat{TG}_{(peak-fast),i} = -0.66 + 0.02*X_{Crossover,\,i} + 0.43*X_{VAT,\,i} + 0.12*X_{HOMAIR,\,i} + 0.23*X_{Aerobic,\,i} - 0.02*X_{SBP,\,i} + \varepsilon_i$$
